# Government policy and agricultural production: a scoping review to inform research and policy on healthy agricultural commodities

**DOI:** 10.1186/s12992-020-0542-2

**Published:** 2020-01-20

**Authors:** Raphael Lencucha, Nicole E. Pal, Adriana Appau, Anne-Marie Thow, Jeffrey Drope

**Affiliations:** 1 School of Physical and Occupational Therapy, Faculty of Medicine, McGill University, 3630 Promenade Sir William Osler, Montreal, QC H3G 1Y5 Canada; 2 Faculty of Sciences, Vrije Universiteit, Amsterdam, Netherlands; 3 Research and Evaluation, PolicyWise for Children and Families, Edmonton, Alberta Canada; 4 Menzies Centre for Health Policy, School of Public Health, University of Sydney, Sydney, Australia; 5 Economic and Health Policy Research, American Cancer Society, Atlanta, USA

**Keywords:** Non-communicable disease, Food policy, Agriculture, Review, Tobacco, Tobacco control, Global health policy

## Abstract

Unhealthy foods and tobacco remain the leading causes of non-communicable disease (NCDs). These are key agricultural commodities for many countries, and NCD prevention policy needs to consider how to influence production towards healthier options. There has been little scholarship to bridge the agriculture with the public health literature that seeks to address the supply of healthy commodities. This scoping review synthesizes the literature on government agricultural policy and production in order to 1) present a typology of policies used to influence agricultural production, 2) to provide a preliminary overview of the ways that impact is assessed in this literature, and 3) to bring this literature into conversation with the literature on food and tobacco supply.

This review analyzes the literature on government agricultural policy and production. Articles written in English and published between January 1997 and April 2018 (20-year range) were included. Only quantitative evaluations were included. Studies that collected qualitative data to supplement the quantitative analysis were also included. One hundred and three articles were included for data extraction. The following information was extracted: article details (e.g., author, title, journal), policy details (e.g., policy tools, goals, context), methods used to evaluate the policy (e.g., outcomes evaluated, sample size, limitations), and study findings. Fifty four studies examined the impact of policy on agricultural production. The remaining articles assessed land allocation (*n* = 25) (e.g., crop diversification, acreage expansion), efficiency (*n* = 23), rates of employment including on- and off-farm employment (*n* = 18), and farm income (*n* = 17) among others. Input supports, output supports and technical support had an impact on production, income and other outcomes. Although there were important exceptions, largely attributed to farm level allocation of labour or resources. Financial supports were most commonly evaluated including cash subsidies, credit, and tax benefits. This type of support resulted in an equal number of studies reporting increased production as those with no effects.

This review provides initial extrapolative insights from the general literature on the impact of government policies on agricultural production. This review can inform dialogue between the health and agricultural sector and evaluative research on policy for alternatives to tobacco production and unhealthy food supply.

## Background

Agricultural production has been deeply transformed by the forces of globalization. On the one hand, export driven agricultural production has significantly increased access to agricultural commodities in inhospitable environments (e.g. the 3 billion bananas consumed in Canada every year [1]). These forces have stimulated the rise in export-oriented crop production in countries around the world. The result has been a concomitant dependence on agriculture-directed foreign investment in exporting countries, and food supply in importing countries. Although theories of comparative advantage point to the benefits of this international supply chain, there are numerous associated problems. These include but are not limited to the negative impact of monocropping [2], including a rise in fertilizer and pesticide use in foreign investment dependant countries [3], dependence on health and environmentally harmful crops such as tobacco [4], enhanced vulnerability to environmental and economic shocks [5], the environmental consequences of extensive refrigeration and transportation emissions across large distances [6], and the pressures on agricultural producing governments to avoid enforcing strong labour and environmental controls for fear of losing revenue from foreign trade and investment (although there is a body of literature suggesting that these standards are actually strengthened through international trade regimes) [7, 8]. These challenges at the intersection of globalization and agricultural production are no more pronounced than in the supply of tobacco and crops used in health-harming foods. Both categories of agricultural production are vulnerable to the above-noted risks and are impacted, and indeed the risks are compounded, by the duel process of efforts to control demand for these products and market instability.

The relationship between government policy and agricultural supply requires analysis on multiple levels. The approaches taken by government to agricultural production are shaped by ideas of economic development, economic interests, the prescriptions and requirements of international agencies (such as the World Bank and the International Monetary Fund) and regimes, local environmental conditions, legacies of national and sub-national institutions among others. Research on agricultural production, policy and public health requires attention to all of these factors and efforts to piece together this puzzle into a comprehensive understanding of how these factors intersect. This review focuses on national level policies and programs as one piece of this puzzle with an attempt to situate these policies in the broader international political economy. As a first step in what is hoped will be greater attention to agriculture and un/healthy commodities as they relate to disease burden and health more generally, this review focuses on the national level recognizing that government policy is one of the more direct and tangible factors shaping agricultural production. The objective of this scoping review is to identify lessons from government policies and programs that have attempted to shift agricultural production in some way, whether this means policies to enhance crop production, induce crop substitution or shift to some other type of employment. Specifically we aim to 1) present a typology of policies used to influence agricultural production, 2) to provide a preliminary overview of the ways that impact is assessed in this literature, and 3) to bring this literature into conversation with the literature on food and tobacco supply. This information will provide a starting point to systematically research how to shape the supply of healthier agricultural commodities and inform policy dialogue to this end.

### Tobacco, food and agriculture

Unhealthy food products and tobacco are two of the leading preventable risk factors for cardiovascular and respiratory diseases and cancer [9, 10]. Demand reduction measures have led to steady but uneven declines in tobacco consumption and are beginning to show impacts on the consumption of unhealthy foods such as sugary drinks [11, 12]. There is also growing recognition of the need to complement these demand reduction measures with attention to issues pertaining to supply. Governments have long been involved in supporting and influencing agricultural production, mainly to support farmer livelihoods and food security. For example, 40% of maize traded on the global market is produced in the United States due to heavy subsidies to maize growers [13]. More recent recognition of the significant cost posed by non-communicable diseases (NCDs) adds an additional public health dimension to this role of government, with a focus on shaping agricultural production in order to foster healthier food supply and reducing harmful products, such as tobacco, in the consumer environment [14]. This global public health imperative needs to be underpinned by research conducted in agriculture-related disciplines, yet there has been little application of findings to public health research questions or policy dialogue across sectors. Understanding this evidence base will be essential for public health policy makers and other stakeholders to formulate effective policy recommendations.

Tobacco and food are important agricultural commodities for many countries and thus agricultural production is tied up with many policy domains and market forces, making it a complex challenge to address through policy and programs [15]. Added to the challenge of controlling production is that if demand remains high, then reductions in production might lead to increases in prices for the commodity, potentially inducing growers to switch back to the production of that commodity. However, production is bound up in the rhetoric of opposition to demand reduction measures by unhealthy product-producing industries such as the tobacco industry [16–18]. A strong evidence base and a deep understanding of the theory and practice of agricultural production by health advocates is a critical part of overcoming likely political and economic challenges.

The main rationale for reducing tobacco production is that tobacco use remains a leading cause of premature preventable death and morbidity globally [19]. Governments have committed, in Articles 17 and 18 of the Framework Convention on Tobacco Control, to actively pursue a policy agenda that supports alternative livelihoods for tobacco farmers, directly and indirectly reducing tobacco supply. Other reasons to reduce tobacco production include the harmful consequences of growing tobacco leaf for the health and economic livelihoods of farmers, as well as for the environment [20–23]. Despite the compelling rationale, implementation of interventions to promote alternative livelihoods has proved challenging. The complex political economy of tobacco production requires comprehensive interventions that address the needs of farmers, from the supply of inputs to market access for alternative crops [24]. Hu and Lee [25] emphasize that “while full-scale crop substitution for tobacco farming … may not be a realistic goal, at least in the near to medium term, encouraging tobacco farmers to shift to other crops has intrinsic benefits … Governments should invest in the infrastructure that will help the farmers grow and market other cash crops” (pg 48).

Food production has experienced massive shifts in the past century with the rise of agricultural technologies, enhanced refrigeration and transportation systems and most importantly the globalization of markets [26]. Global agriculture trade accounts for over 20% of global calorire production [13]. This shift has led to shifts from subsistence to export-driven crop production, which in turn has led to the homogenization of crop production [27]. This homogenization has reduced biological diversity in the food system, and “the global agricultural system currently overproduces grains, fats, and sugars while production of fruits and vegetables and protein is not sufficient to meet the nutritional needs of the current population” [28]. Although this shift has been credited with helping to reduce rates of global hunger, the substitution of nutrient rich crops for wheat, rice and maize has contributed to both undernutrition and obesity with concomitant increases in rates of cardiovascular disease and diabetes, particularly in low- and middle-income countries (LMICs) [29–32]. For example, the rise in rates of diabetes in India has been attributed to the move away from high-density, protein-rich legumes towards rice and wheat [32]. Negin and colleagues provided an overview of the shifts in agriculture production in Asia and highlight that in India, “many of the secondary food grains such as pulses, which are major sources of protein in vegetarian Indian diets, as well as millets such as sorghum, pearl millet, and finger millet, which serve as staples in dryland areas and are rich in micronutrients, were underemphasized” [33]. This is a common observation in countries around the world and has led to an emerging consensus that agricultural production requires another major shift towards greater production of fruits and vegetables and nutrient-rich cereals and pulses [34].

## Methods

### Research question

The research question informing this scoping review is: What types of government policies and programs facilitate changes in agricultural production? To answer this question we categorize the types of: 1) policies and programs governments have used to encourage farmers to *move out* of growing a particular crop, 2) policies and programs used to encourage farmers to *move into* growing a particular crop, 3) research methodologies used to assess these policies and programs and finally to 4) outcomes used to evaluate the policies and programs and finally 5) summarize the impact that these interventions have had on agricultural production.

### Search strategy

We employ a scoping review methodology using methods developed by Arksey and O’Malley. The main aim of a scoping review is to provide an overview of published literature in order to identify key trends, approaches used to study a topic or gaps in an area of interest. This approach is distinct from a systematic review that attempts to pool and analyze data from existing studies in order to draw stronger conclusions on a measurable topic of interest. Arksey and O’Malley characterize a scoping review as a type of research synthesis that broadly maps the current literature on a certain topic [35]. Key terms and concepts were developed from previously identified articles on the topic and were grouped into farmer behavior (e.g., “crop diversification”, “off-farm labour migration”) and policy categories (e.g., “subsid*”, “price control*”). A university librarian with expertise in the field of agriculture was consulted for input on key terms and database selection. For complete search terms please refer to Table 1. The electronic databases SCOPUS and CAB Abstracts were used to locate articles. The search was completed over a three-month period between March and June 2018. We included articles written in English and published between January 1997 and April 2018 (20-year range). The time frame was sufficient to capture policies and programs implemented in the era of neoliberalism, such as privatization along the supply chain, the elimination of quantitative restrictions on trade and the reduction of other barriers to trade such as tariff reductions, among other policy shifts, which dramatically shaped the governance of agricultural production [36]. We did not place any restrictions based on geography. Studies from all countries or region were eligible for inclusion.

**Table 1:** Search terms

Search Terms	
(evaluat* OR assess* OR analy*) AND (government OR polic* OR “non-governmental organization” OR ngo OR “government program*”) AND (agricultur* OR farm*) AND Farmer Behavior: (“crop diversification” OR “crop alternative*” OR “crop substitut*” OR “crop change” OR “off-farm migrat*” OR “off-farm labor migrat*” OR “occupational migrat*” OR “labor migrat*” OR “farm* deci*”) OR Policy Categories: (subsid* OR “price support*” OR “input support*” OR “tax concession*” OR quota* OR tariff* OR “import control*” OR “crop control*” OR “credit support*” OR “price control*” OR “export support*”)	

### Article selection

Inclusion criteria consisted of articles that quantitatively evaluated a program or policy that affected farmer decisions, for example, a program providing subsidized seeds to grow a certain crop. Only articles that present empirical research were included; mainly quantitative evaluations, although some studies collected qualitative data (e.g. focus groups) to supplement the quantitative analysis. We made this decision to focus on evaluation researc in order to identify general patterns of policy and program impact. This decision also contributes to our ability to draw methodological lessons (e.g. common outcomes and evaluation methods) for future research that attempts to measure the impact of policy and programs on tobacco and food supply.

**Fig. 1:**
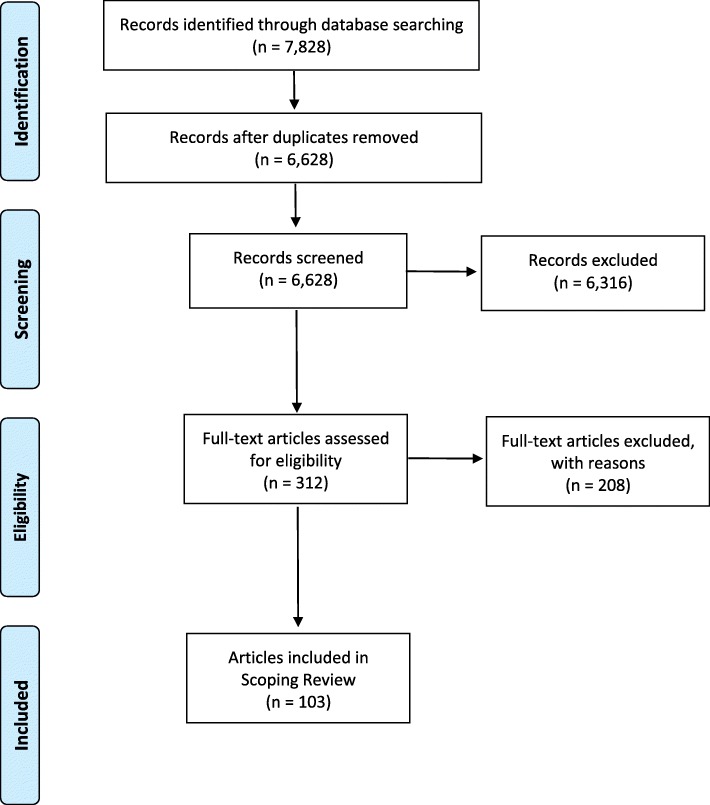
Modified PRISMA Flowchart

This scoping review strategy involved an initial review of article titles. If the title did not provide sufficient information, the abstract was then reviewed. Six thousand three hundred and sixteen articles were excluded at this stage. Duplicates were also removed at this stage. Article abstracts were then reviewed for full-text consideration. For full-text selection, three authors (RL, NP, AA) assessed agreement on the interpretation of the inclusion criteria by independently reviewing the full text of 10 articles. This review resulted in 100% agreement on whether the article would be included or excluded. One member continued to review the remainder of articles for full-text selection, discussing any challenges with the research team throughout. Figure 1 illustrates the search and selection process using a modified PRISMA flow diagram [37].

### Data extraction

A data-extraction table was developed by the lead author and NP. The data extraction categories were informed by the overarching research question. The following information was extracted from the included articles: article details (e.g., author, title, journal), policy details (e.g., policy tools, goals, context), methods used to evaluate the policy (e.g., outcomes evaluated, sample size, limitations), and study findings. Two authors (RL, NP) independently reviewed and extracted information from three articles, then compared results. Each article had a possible score of 22 for agreement (number of data columns) with a possible total score of 66 for all three articles. The total agreement score was 63/66 or a 95% agreement rate. Discrepancies between the two raters were resolved by a third member of the research team (AA). One member continued to extract the remainder of the articles.

## Results

### Descriptive results – policy characteristics

One hundred and three articles were included for full text review. Totals for policy characteristics will differ due to studies being classified under more than one category, or due to studies not reporting on all characteristics. As some articles did not report on all descriptive categories, the following proportions are from those reported. The term intervention will be used to refer to both policies and programmes. Articles were categorized into four intervention types, (1) input support, (2) output support/restriction, (3) technical support, and (4) financial support. Table 2 presents definitions of the policy types.

**Table 2:** Policy Type Definitions

Policy Types	
Type 1: Financial Support	Financial aid provided to farmers in the form of credits, tax benefits, loan aid, insurance aid or financial incentives
Type 2: Input Support	Materials provided to farmers to aid in production in the form of subsidized seeds, fertilizer or machinery
Type 3: Output Support/Restrictions	Aid for or restrictions on farmers regarding post-production activities, such as supply chain support, price supports, price controls, production quotas
Type 4: Technical Support	Aid provided to farmers in the form of extension services, investment in structural development (e.g., road construction, rural development), or in the organization of farming cooperatives

### Descriptive results – policy evaluation

Fifty-four studies assessed production. Of these studies 10 collected primary data through surverys and interviews, 10 studies used both primary and secondary data and the remaining 34 studies analyzing secondary data. The articles identified in this scoping review assessed a wide range of outcomes. We categorized the outcomes for clarity. The remaining articles assessed land allocation (*n* = 25) (e.g., crop diversification, acreage expansion), efficiency (*n* = 23), rates of employment including on- and off-farm employment (*n* = 18), and farm income (*n* = 17). Other outcomes were measured, such as exports, production costs, economic growth, and number of farms, each composing less than 5% of the included studies, but together composing 13% (*n* = 20) of the total. Crops targeted by a policy at times differed from crops targeted by the study. The agricultural product most used to evaluate a policy were cereal crops (37%, *n* = 39), followed by oilseed crops (8%, *n* = 8), livestock (8%, *n* = 9), and dairy (8%, n = 9). Less common were agricultural commodities such as fruits, vegetables, and legumes (e.g., beans, pulses) each comprising less then 5% of the studies but together represented 39% (*n* = 41) of the total. Thirty percent of the evaluations took place less than 2 years after the intervention (*n* = 29) and another 30%, 6–10 years after the intervention. A full list of countries by class is listed in Additional file 1, and a full bibliography of included studies is provided in Additional file 2. For complete information on policy evaluation descriptive information please refer to Table 3 and Table 4.

**Table 3:** Policy Descriptives

Descriptives	
Policy Types, *n (%)*	
Financial Support	65 (47%)
Input Support	41 (29%)
Output Support/Restrictions	18 (13%)
Technical Support	15 (11%)
Total	139^a^
Funding Source, *n (%)*	
Government	99 (82%)
Donor Agencies	8 (7%)
Foreign Organization	6 (5%)
Foreign Government	6 (5%)
Total	119
Policy Reach, *n (%)*	
National	59 (57%)
Multinational	36 (35%)
Other^b^	8 (10%)
Total	103
Location, *n (%)*	
European Union	34 (32%)
China	15 (14%)
United States	9 (9%)
Other^b^	47 (44%)
Total	105
Year Implemented, *n (%)*	
> Year 2000	68 (60%)
< Year 2000	45 (40%)
Total	113
Target Crop, *n (%)*	
Cereal Crops (e.g., wheat, maize, rice)	31 (51%)
Bioefuel/Oilseed Crops (e.g., castor oil, soybean oil, cottonseed oil)	5 (8%)
Other^b^	25 (41%)
Total	61

^a^Groups ≤ 5%

^b^Totals will vary due to studies being classified under more than one category, or not reporting on all characteristics

**Table 4:** Study method descriptives

Descriptives	
Data Type, *n (%)*	
Primary Data	17 (17%)
Secondary Data	69 (67%)
Combination	17 (17%)
Total	103
Data Collection Method, *n (%)*	
Survey	93 (79%)
Economic Reports	13 (11%)
Interviews	9 (8%)
Focus Groups	2 (2%)
Total	117
Study Outcomes, *n (%)*	
Production	54 (34%)
Land allocation (e.g., crop diversification, acreage expansion)	25 (16%)
Efficiency	23 (15%)
Employment	18 (11%)
Farm Income	17 (11%)
Other^a^	20 (13%)
Total	157
Study Location, *n (%)*	
China	14 (13%)
United States	9 (8%)
The Czech Republic	6 (6%)
Other^a^	79 (73%)
Total	108
Study Target Crop, *n (%)*	
Cereal Crops	39 (37%)
Oilseed Crops	8 (8%)
Livestock	9 (8%)
Dairy	9 (8%)
Other^a^	41 (39%)
Total	106
Time Period of Evaluation, *n (%)*	
≤ 2 Years	29 (30%)
3–5 Years	18 (18%)
6–10 Years	29 (30%)
11–20 Years	16 (16%)
≥ 21 Years	6 (6%)
Total	98

^a^Totals will vary due to studies being classified under more than one category, or not reporting on all characteristics

### Policy context

Policy and programs are established in particular political, social and economic contexts. Understanding context can contribute to undersanding policy implementation, uptake and impact by identifying policy levers, obstacles and windows of opportunity. We gathered data on the reported economic, political, environmental or social circumstances that were reported as a factor contributing to a change in policy or program.

**Fig. 2:**
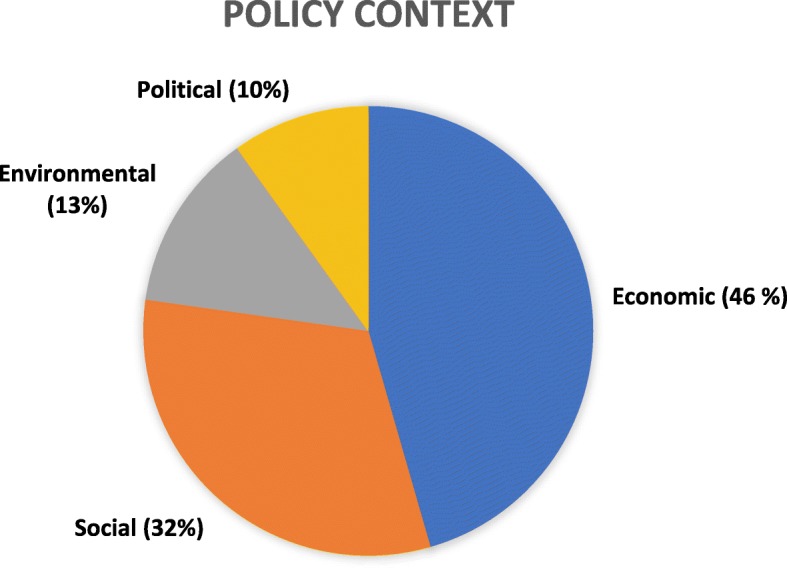
Contextual factors shaping government policy

Proportional representation of the context categories reported by the identified articles can be found in Fig. 2. Many studies reported issues of food security and crop production as a prominent factor contributing to intervention. For example, countries in sub-Saharan Africa have introduced input support programs to address this challenge. Nigeria, for example, found their consumption of rice far exceeded domestic production and the country was relying on costly imports. This situation prompted the government to provide input support measures to farmers such as high-yield seedlings, fertilizers and herbicides to increase the production of rice [38]. A global shift towards market liberalization and away from trade-distorting policies was also mentioned by a handful of studies. For example, in 1996, the United States introduced decoupled payments (i.e., annual subsidies no longer linked to crop production) aimed at decreasing government involvement in farming decisions, and instead gearing farmers towards more market-oriented behavior [39].

Policy change attributed solely to political circumstances (as opposed to economic and social, for example) were reported less than others. Of those reported, many involved countries acceding to the EU and thus becoming eligible for the EU Common Agricultural Policy support measures, such as Poland in 2004 and Bulgaria in 2007 [40, 41]. In Poland for instance, there was an increase in agricultural income in the first few years following their integration with the EU. Change in government control is another example of a political situation contributing to policy change, such as in the case of West Bengal, India. In 1977, the Left Front Coalition headed by the Communist party of India was voted into power and shortly after, implemented a range of development and welfare policies, one of which was an agricultural input support program of subsidized seeds, fertilizers and pesticides [42].

Many policies were enacted as a response to social circumstances, primarily concerning the welfare of farmers. For example, after identifying access to agricultural inputs as a leading challenge for farmers, Zimbabwe implemented a subsidy programme that provided credits via e-vouchers to farmers for purchasing inputs, as well as coordinated with input suppliers to ensure adequate stock levels and fair prices [43]. Lastly, environmental conditions have increasingly become a prominent driver for policy change. Due to a rising demand for sustainable fuel sources, governments are introducing policies encouraging the production of bioefuel crops. In 2003, the European Union set targets for the proportion of bioenergy in total energy demand. It was up to each country on how to achieve the targets using methods such as tax exemptions and production quotas, all of which were expected to encourage farmers to allocate more acreage to biofuel crops [44]. Similar to the EU, Brazil in 2004 implemented country-wide targets for biodiesel output. To achieve these goals Brazil used policy tools such as tax and credit incentives for biofuel producers, as well as increased per-sack prices for farmers growing biofuel crops [45].

### Impact

#### Policy type one – input support

A summary of the impact of the four policies examined is presented in Table 5. Sixteen studies estimated the impact of input support on production (yields and productivity). In nine of these studies [42, 46–53], input support such as seeds, fertilizer and equipment subsidies, and provision of improved and high quality seeds resulted in an increase in agricultural production. For example, the Malian Fertilizer Subsidy Programme allows farmers to purchase subsidized fertilizer from authorized distributers with the goal of increasing national agricultural production. To evaluate this policy, Theriault et al. (2018) assessed maize and sorghum yields for those who participated in the programme as compared to those who did not participate in the programme and found significantly higher yields for those who participated [54]. Two other studies that examined the impact of providing input support found a negative association between input support and production [55, 56]. This negative association was attributed to redundancy and inefficient use of resources in the subsidy program [55] and a lack of data on the amount of actual subsidies received [56]. Other studies identified that the provision of poor quality inputs may account for the lack of effect of input support programs on production [57]. One study estimated the impact of reducing input subsidies on agricultural production and found that this resulted in lower productivity [58]. Possebom (2017) on the other hand examined the spillover effect of reducing import tariffs on industrial inputs on other economic sectors. The creation of free trade zones and reduction in import tariffs on industrial inputs led to a decrease in agricultural total production per capita indicating a negative spillover effect of this industrialization policy on the agricultural sector. Four studies found no effect of input subsidies on production [59–62]. Also, four studies [43, 51, 63, 64] demonstrated that providing improved subsidized seed and agricultural inputs such as fertilizer led to an increase in farmers’ income. Furthermore, three of the four studies that examined the effect of input support on off-farm employment found a positive impact while one found a negative impact on off-farm employment.

**Table 5:** Number of studies demonstrating impact based on policy type

Selected Outcomes Measured	Input Support			Output Support/Restriction			Technical Support			Financial Support		
(↑ = Increase, ↓ = Decrease)	Positive	Negative	No Effect	Positive	Negative	No Effect	Positive	Negative	No Effect	Positive	Negative	No Effect
↑ Production	9	5	4	6	3	1	4	2	4	9	7	12
↑ Net Profit	2						2			3		
↑ Farmer Income	4	1		1			5	1	1	11	4	2
↑ Crop Diversification	1	2		2	1					1		
↑ Land Allocated to farming	1	1	2			1				6	1	1
↑ Off-farm Employment	3	1					1	1	1	5	1	3
↑ Yield	6		1				2			3		1
↑ Land not allocated to farming	1									2		
↑ On-farm Employment	1			1			1		1	6	6	3
↓ Poverty Severity	1											
↓ Relative Deprivation	1											
↑ Exports	1			1							1	1
↑ Productivity (output/hectare)	1						3			1		
↑ Farm Size			1				3			1		2
↑ Efficiency	1		1							8	14	5
↑ Land allocated to one crop from another	2		1		1	1	1			3		2
↑ Number of Farms							2			5		1
↑ Proportion of Livestock							2			5		

#### Policy type two – output support/restriction

Price supports, such as counter cyclical payments and price incentives, were shown to increase production and crop diversification [65, 66]. For example, Alia et al. (2017) assessed the impact of Benin’s price support policies on cotton production. The policy functioned by government increasing producer prices for cotton by 5% initially and subsequently by 25%. Statistical analysis presented showed that this price support was associated with an increase in cotton supply, as the stability of the crop price encouraged more farmers to grow cotton [65]. Two studies evaluated the effect of market liberalization on commodity of interest and a substitute commodity on production. For instance, Fraser (2006) found that reducing import tariffs (15% reduction) and later elimination of these tariffs on fruits and vegetables resulted in a reduction in fruit and vegetable production from 15,142 hectors to 13,365 hectors in the local market [67]. However an opposite policy of increasing import tarrifs on powdered milk by 40% (a substitute for milk) in addition to price supports for milk led to an increase in the production of 37% [46]. It is important to note that other contextual factors may affect the impact of policies such as trade liberalization. For example, Fraser (2006) found that international trade liberalization contributed to the geographic move of the vegetable and fruit processing industry. As a result, local producers no longer had a market for processed vegetables which may account for the reduction in vegetables and fruits production observed.

#### Policy type three – technical support

Ten studies evaluated the effect of technical support on production. Technical support captured a wide range of policy tools such as extension services (e.g., government sending service workers sharing farming knowledge and techniques with farmers), investment in structural development (e.g., road construction, rural development), and support in the establishment of farming cooperatives. Once again, production and farmer income were the outcomes most evaluated. The impact of extension services on production were mixed; three studies [47, 49, 60] reported an increase in production, and four a decrease or no effect on production [50, 57, 62, 63]. Higher frequency, quality of services provided, on field practicals and well trained extensions officers were factors associated with a positive impact of this policy on production. Ross (2017) investigated this relationship using an experimental design. One group of farmers was provided with agricultural inputs at a subsidized price, another group was provided with the same package in addition to extension services including soil fertility management, legume production, in-organic fertilizer and farm management methods, and the third control group received no support. It was found that both groups that received an intervention increased total household output, however only the group who received both subsidies and extensions support had statistically significant results [68].

Investment in structural infrastructure such as roads and forming of farmer cooperatives were also shown to increase farm incomes [45, 47, 63, 69]. Combining extension support with access to subsidized inputs (e.g., seeds, fertiliser) was found to be a commonly implemented and effective approach. For example, the Native Tobacco Intensification Program in Indonesia, the Agriculture Input Support Programme in Zimbabwe, and the Homestead Food Garden Programme in South Africa all paired input support tools such as garden equipment, seeds, and fertilizer with extension support such as training sessions on farming techniques and optimization methods. The studies evaluating these policies all reported increases in the production of their targeted crops, tobacco, tomatoes and maize, respectively [43, 47, 70]. With the exception of one study, all studies evaluating the effect of extension services, infrastructure and or farmer corporatives on income or farm size reported an increase in farmer incomes or farm size.

#### Policy type four – financial support

Financial support was the most commonly evaluated intervention. Financial support included cash subsidies, credits, tax benefits, loan aid and insurance aid. Whereas all three of the previous policy type categories were associated with increases in crop production, financial support had an equal number of studies reporting increased production as studies finding no effects. Also out of 11 studies that examined the impact of financial support on farmer’s income and profit, three found a negative association. Furthermore, there is evidence to suggest that the impact of financial support on farmer’s income or revenues is diverse and dependent on factors such as farm size and production capacity. For example, Naglova and Gurtler (2016) found that direct payments improved the farm income and revenue of medium and large scale farms but had a negative impact on smallscale farmer’s income. According to Judzinska (2013), the diverse impact of direct payments could be as a result of the fact that direct payment supports farmers economically and gives them the opportunity to increase their production capacity but at the same time could discourage farmers from improving farm efficiency. This was evident in the fact that out of 27 studies that examined the effect of financial support on efficiency, 19 found negative or no effect of financial support on efficiency. For example, Direct Income Transfers provided to Greek olive producers as part of the Common Agricultural Policy (CAP) of the European Union were found to have a negative impact on efficiency, indicating that an increase in payments led to a decrease in efficient farming [71]. Financial support policies have also been associated with shifts in land allocation such as increases in land allocated to farming, number of farms, crop specialization, and farmer participation. For example, Galluzzo evaluated a range of CAP direct payments and found that the Single Area Payment Scheme had a positive impact on crop specialization, or choosing to grow a certain crop over others [72].

## Discussion

This scoping review identified 103 studies that evaluated the impact of input support, output support/restriction, technical support, and financial support on agricultural outcomes. This review finds that much can be accomplished at the national level to shape agricultural production, but the national context is tightly bound to global political and economic factors. First, we found that input supports, such as subsidies on fertilizers, seeds or farm equipment, generally resulted in positive changes in production and farm income. This finding corresponds with research in the tobacco control literature that finds that inputs are a key factor in farmers’ decision to enter into contract with leaf buying companies [22, 23]. Second, these findings also point consistently to the high level of importance of education and support for farmers, most often in the form of extension services. The studies that evaluated the impact of education support found positive increases in such outcomes as production and income. This finding also corresponds with cross-sectional studies in which tobacco farmers identified receiving extension services as extremely important in supporting their production and livelihoods [73, 74]. Third, the findings from this review suggest that price support mechanisms have led to increases in production.

The findings that demonstrate a positive impact of input supports are consistent with general policy shifts away from public support in the agricultural sector (and other public sectors). The absence of input support from government both contributes to and is a result of smallholder farmers entering into contract with private companies. These contractual relationships can improve production but also concentrate power with private entitites who then determine the quantity of product purchased, have power to evaluate the quality of the commodity and ultimately the price paid to the farmer [75, 76]. Such contracts often involve inflated prices for inputs and reduced prices offered for the commodity at market [77, 78]. Tobacco leaf-buying companies can attract farmers to enter into contracts under these unfavourable conditions because the arrangement facilitates easier access to inputs, and sometimes also cash loans, particularly when more traditional credit is scarce [79]. It must be recognised that such private investment is also likely to limit the operationalization of government efforts to increase production of healthy agricultural commodities. For example, where governments have withdrawn from providing extension services for tobacco, private companies have taken over [73, 74, 80]. In Kenya, the agricultural ministry does not provide input supports or extension services to tobacco because the government has listed tobacco as an unscheduled crop, thus taking a hands-off approach to tobacco production [73]. Farmers report that services provided by tobacco companies are often of high quality and they believe that this support contributes to improved yields. This suggests that governments may need to examine opportunities to curtail private sector investment in tobacco if alternatives are to be meaningfully pursued.

What complicates this dynamic of government involvement in providing input support or subsidies and extension services to smallholder farmers is the general guidance by key international agencies such as the International Monentary Fund (IMF) for governments to remove subisidies and other public supports. Daoud and colleagues [81] conducted a comprehensive review of IMF policies and found that there is a general push for government to remove subsidies although the extent of implementation of this guidance is less clear. Meurs and colleagues [82] confirm that IMF policy guidance has encouraged or even compelled the reduction of government expenditure in the agricultural sector in their analysis of the place of IMF policy in Uganda, Tanzania and Malawi. For example, in Malawi they find that “policy measures to cut back expenditures include reducing the budget for maize procurement and agricultural subsidies” among other reductions in public spending. There are important implications stemming from such market-oriented measures that require further study. What seems clear is that such market-oriented measures have created a situation where government support for agricultural production will require deeper ideological shifts in the relationship between government and market [83].

Certainly if governments are to move towards promoting agricultural commodities from the standpoint of health and environmental sustainability there will be a need to develop robust markets for a wider range of commodities. It is an uncontroversial fact that commodities like tobacco or sugar are attractive to farmers because of a combination of factors such as access to markets, contractual arrangements that allow access to inputs and loans, and other facilitators along the supply chain [84–86]. For example, Natarajan points out that tobacco farmers in South India grow the crop due to its amenability to the environment and the lack of profitable alternatives [87]. Similarly, studies in Malawi and Kenya also find that farmers continue to grow tobacco, despite limited income, due to a perceived lack of alternatives [22, 23]. This review provides important direction for research on alternatives. For example, the basic framework presented in this review illustrates the different outcomes that can be examined such as production levels, income, and land allocation. In addition, there are certain policies that demonstrate patterns of effectiveness across different contexts and crops, such as input supports, extension services, and price supports. There is a need to examine how each policy impacts production and farmer decisions and how outcomes are impacted by combined policy approaches. Experiments that attempt to shift agricultural production away from tobacco and towards healthy food crops can begin with this typology. As we noted earlier, in addition to the policies themselves, there is a continued need to situate these policies in the broader political economy and to analyse the processes of policy development and implementation. There is clear evidence that trade and investment regimes have fostered consumer access to products such as tobacco and unhealthy foods and beverages [88, 89]. These regimes have also facilitated market access and corresponding influence over policy space by these industries. There remains a need to extend the analysis along the supply chain to examine how such regimes shape production of basic agricultural commodities, and how these regimes interconnect with what is happening at the national and sub-national levels.

The aim of this review is to contribute to future policy and research to affect the supply of healthier agricultural products, including in relation to the pressing need to shift support away from tobacco and unhealthy food crops and towards healthy food crops [90]. In particular, the findings can inform strategic and informed advocacy by health actors, as the policies identified in this review reflect the core global approaches to agricultural investment. These policy and programs fall under the purview of agricultural or other ministries with economic development portfolios, and will thus require sensitization of health sector actors to communicate the benefits of intervention in the agricultural supply chain for the purpose of health promotion and disease prevention. This reflects previous research indicating that a coherent approach to healthy agricultural product production will involve strategic engagement across ministries [91, 92].

This review also suggests that there is a strong evidentiary basis for public health advocacy for input support, provision of extensions services and financial support to increase production of healthy food, and government disinvestment away from tobacco and towards alternative crops. It is important to develop an understanding of the agricultural policy context. As our review indicates, the role of government in agricultural markets has shifted dramatically since the beginning of the neoliberal era [83]. This shift in some ways has distanced government from direct involvement in the provision of extension and other supports. Policy interventions targeting agriculture are complicated by decades-long shifts in government withdrawal from market activities driven by the neoliberal policy paradigm, and the concomitant primacy of economic considerations in agricultural decision making. The result of this has been the over-privileging of the role of the private sector either at the expense of government participation in the market or perhaps more commonly reorienting government resources to serve these private interests often at the expense of smallholder farmers [93, 94]. For example, in Zambia the push for value-addition along the agricultural supply chain, in the absence of a government policy to reduce the tobacco supply, led to government support for tobacco processing and manufacturing [80, 95]. This economic decision will likely lead to increased consumption of tobacco leaf in Zambia, contrary to public health objectives. Therefore health advocates must engage with this context to understand what governments can and cannot do along the supply chain and what types of policies they are more likely to pursue.

Emerging research on alternatives to tobacco has demonstrated that sustained shifts in production require deep integration with viable alternative markets [20, 96, 97]. It will be important to evaluate not only the farm-level indicators such as production and income, but also broader political economic factors such as market access and trade and investment regimes [15, 98].

## Limitations

This review has a number of limitations common to the scoping review methodology. It is possible that the use of additional literature data bases would have yielded further articles. However, given the involvement of a specialist librarian it is anticipated that the two data bases chosen were appropriate to capture the breath of research on this topic. Because of the broad scope of the review we did not have the financial resources to extend the search and analysis to the grey literature. There are certainly reports published by government, nongovernmental and intergovernmental agencies that are relevant to this topic. It is hoped that future work in this area will draw from these publications. The quality of the methods used in the included studies was not systematically analyzed. However, because the purpose of this review was to identify the breadth of research in this field in order to inform future, more targeted, research on interventions to shape the tobacco and food supply, we think our approach achieved this end. The relationship between policy and agricultural production may be context dependant and the contextual nature of this relationship requires further systematic examination to determine the policies that are effective or ineffective across contexts. Last, this review sought to bring the general agricultural literature into conversation with the public health literature on tobacco and food production. However, because different crops have different end uses it is important that future research rigorously seeks to understand how demand shapes supply. Here we included crops such as rice, wheat, and others that likely differ greatly from tobacco given the inelasticity of demand.

## Conclusions

There is a need to apply existing knowledge of effective interventions targeting agricultural production and farm level economic factors. Evaluation studies suggest that certain types of interventions are more effective than others. There is also a need to conduct rigorous evaluation studies on interventions specifically aiming to shape the tobacco and food supply. To date, such research remains scarce.

## Supplementary information

**Additional file 1: Table S1.** Countries represented in the evaluation research by class.

**Additional file 2.** Full bibliography of included literature.

## Data Availability

The datasets used and/or analysed during the current study are available from the corresponding author on reasonable request.
