# Supplementary material for: Government policy and agricultural production: a scoping review to inform research and policy on healthy agricultural commodities
Source: Global Health. 2020 Jan 20;16:11. doi: 10.1186/s12992-020-0542-2 (PMC6971899; doi:10.1186/s12992-020-0542-2)
Supplement: Supplementary file 1 — Additional file 1: Table S1. Countries represented in the evaluation research by class. [file 12992_2020_542_MOESM1_ESM.docx]

**Table __. Country Classes (Study Method Descriptives)**

| **Country** | **Region** | **Class** | **Count** | |
| --- | --- | --- | --- | --- |
| Albania  Bangladesh  Benin  Brazil  Bulgaria  Canada  China  Czech Republic  Denmark  Democratic Rep. of Congo  European  Union  France  Germany  Ghana  Greece  Guyana  Hungary  India  Indonesia  Iran  Ireland  Italy  Kazakhstan  Latvia  Malawi  Mali  Moldova  Mongolia  Netherlands  Nigeria  North Macedonia  Paraguay  Poland  Romania  Senegal  Serbia  Slovenia  South Africa  Spain  Sri Lanka  Sweden  Thailand  Uganda  United States  Uruguay  Zambia  Zimbabwe  **Total** | Europe & Central Asia  South Asia  Sub-Saharan Africa  Latin America & Caribbean  Europe & Central Asia  North America  East Asia & Pacific  Europe & Central Asia  Europe & Central Asia  Sub-Saharan Africa  Europe & Central Asia  Europe & Central Asia  Europe & Central Asia  Sub-Saharan Africa  Europe & Central Asia  Latin America & Caribbean  Europe & Central Asia  South Asia  East Asia & Pacific  Middle East & North Africa  Europe & Central Asia  Europe & Central Asia  Europe & Central Asia  Europe & Central Asia  Sub-Saharan Africa  Sub-Saharan Africa  Europe & Central Asia  East Asia & Pacific  Europe & Central Asia  Sub-Saharan Africa  Europe & Central Asia  Latin America & Caribbean  Europe & Central Asia  Europe & Central Asia  Sub-Saharan Africa  Europe & Central Asia  Europe & Central Asia  Sub-Saharan Africa  Europe & Central Asia  South Asia  Europe & Central Asia  East Asia & Pacific  Sub-Saharan Africa  North America  Latin America & Caribbean  Sub-Saharan Africa  Sub-Saharan Africa | Upper Middle Income  Lower Middle Income  Low Income  Upper Middle Income  Upper Middle Income  High Income  Upper Middle Income  High Income  High Income  Lower Income  High Income  High Income  High Income  Lower Middle Income  High Income  Upper Middle Income  High Income  Lower Middle Income  Lower Middle Income  Upper Middle Income  High Income  High Income  Upper Middle Income  High Income  Low Income  Low Income  Lower Middle Income  Lower Middle Income  High Income  Lower Middle Income  Upper Middle Income  Upper Middle Income  High Income  Upper Middle Income  High Income  Upper Middle Income  High Income  Upper Middle Income  High Income  Upper Middle Income  High Income  Upper Middle Income  Low Income  High Income  High Income  Lower Middle Income  Lower Middle Income | | 1  2  2  2  2  1  14  6  1  1  4  4  3  4  2  1  1  3  1  1  1  1  1  2  4  1  1  1  2  3  1  1  3  1  1  1  2  1  2  1  4  1  1  9  1  4  1  **108** |
