# Supplementary material for: Government policy and agricultural production: a scoping review to inform research and policy on healthy agricultural commodities
Source: Global Health. 2020 Jan 20;16:11. doi: 10.1186/s12992-020-0542-2 (PMC6971899; doi:10.1186/s12992-020-0542-2)
Supplement: Supplementary file 2 — Additional file 2. Full bibliography of included literature. [file 12992_2020_542_MOESM2_ESM.docx]

1. Aditya KS, Subash SP, Praveen KV, Nithyashree ML, Bhuvana N, Sharma A. Awareness about Minimum Support Price and Its Impact on Diversification Decision of Farmers in India. Asia and the Pacific Policy Studies. 2017;4(3):514–26.

2. Adjimoti GO, Kwadzo GTM, Sarpong DB, Onumah EE. Input policies and crop diversification: evidence from the Collines Region in Benin. African Development Review. 2017;29(3):512–23.

3. Ajani RO, Oluwasola O. Appraisal of upland rice production in southwestern Nigeria: a Policy Analysis Matrix approach. Journal of AgriScience. 2014;4(8):399–408.

4. Alia DY, Floquet A, Adjovi E. Heterogeneous Welfare Effect of Cotton Pricing on Households in Benin. African Development Review. 2017;29(2):107–21.

5. Andri KB, Santosa P, Arifin Z. An empirical study of supply chain and intensification program on Madura tobacco industry in East Java. Journal of Agricultural Research. 2011;6(1):58–66.

6. Ayouba K, Boussemart JP, Vigeant S. The impact of single farm payments on technical inefficiency of French crop farms. Review of Agricultural, Food and Environmental Studies. 2017;1–23.

7. Bahta YT, Owusu-Sekyere E, Tlalang BE. Assessing participation in homestead food garden programmes, land ownership and their impact on productivity and net returns of smallholder maize producers in South Africa. Agrekon. 2018;57(1):49–63.

8. Bakucs LZ, Latruffe L, Ferto I, Fogarasi J. The impact of eu accession on farms’ technical efficiency in Hungary. Post-Communist Economies. 2010;22(2):165–75.

9. Bardhan P, Mookherjee D. Subsidized farm input programs and agricultural performance: A farm-level analysis of West Bengal’s green revolution, 1982-1995. American Economic Journal: Applied Economics. 2011;3(4):186–214.

10. Benin S. Impact of Ghana’s agricultural mechanization services center program. Agricultural Economics (United Kingdom). 2015;46:103–17.

11. Bojnec S, Latruffe L. Farm size, agricultural subsidies and farm performance in Slovenia. Land Use Policy. 2013;207–17.

12. Burns CB, Prager DL. Does crop insurance influence commercial crop farm decisions to expand? An analysis using panel data from the Census of Agriculture. Journal of Agricultural and Resource Economics. 2018;43(1):61–77.

13. Capitanio F, Gatto E, Millemaci E. CAP payments and spatial diversity in cereal crops: an analysis of Italian farms. Land Use Policy. 2016;574–82.

14. Chibwana C, Fisher M, Shively G. Cropland Allocation Effects of Agricultural Input Subsidies in Malawi. World Development. 2012;40(1):124–33.

15. D’Antoni JM, Mishra AK, Barkley AP. Feast or flee: Government payments and labor migration from U.S. agriculture. Journal of Policy Modeling. 2012;34(2):181–92.

16. D’Antoni JM, Mishra AK, Blayney D. Assessing participation in the milk income loss contract program and its impact on milk production. Journal of Policy Modeling. 2013;35(2):243–54.

17. Dai X, Pu L, Rao F. Assessing the effect of a crop-tree intercropping program on smallholders’ incomes in rural Xinjiang, China. Sustainability (Switzerland) [Internet]. 2017;9(9). Available from: https://www.scopus.com/inward/record.uri?eid=2-s2.0-85028728410&doi=10.3390%2fsu9091542&partnerID=40&md5=3be3fdb7e14eb657eb1afa2a101bd0a8

18. Dong SK, Li HJ, Li YY. Social-ecological impact evaluation on eco-environmental policies associated western china development. 2010;2:1361–7.

19. Doucha T, Stolbova M, Lekesova M. Assessment of support for farms in the Czech less favoured areas with special regards to cattle breeding. European Countryside. 2012;4(3):179–91.

20. Fraser EDG. Crop diversification and trade liberalization: linking global trade and local management through a regional case study. Agriculture and Human Values. 2006;23(3):271–81.

21. FuJin Y, DingQiang S, YingHeng Z. Grain subsidy, liquidity constraints and food security-impact of the grain subsidy program on the grain-sown areas in China. Food Policy. 2015;114–24.

22. Gafar J. Economic policy and growth: agricultural performance and the rice industry of Guyana. Journal of Development Studies. 1998;19(1):153–74.

23. Galluzzo N. Analysis of subsidies allocated by the Common Agricultural Policy and cropping specialization in Romanian farms using FADN dataset. Scientific Papers Series - Management, Economic Engineering in Agriculture and Rural Development. 2016;16(1):157–64.

24. Galluzzo N. Analysis of some economic variables in Slovenian farms using FADN dataset. Scientific Papers Series - Management, Economic Engineering in Agriculture and Rural Development. 2017;17(1):215–21.

25. Galluzzo N. A non-parametric analysis of technical efficiency in bulgarian farms using the fadn dataset. European Countryside. 2018;10(1):58–73.

26. Gardebroek C, Reimer JJ, Baller L. The Impact of Biofuel Policies on Crop Acreages in Germany and France. Journal of Agricultural Economics. 2017;68(3):839–60.

27. Goodwin BK, Mishra AK. Another look at decoupling: additional evidence on the production effects of direct payments. Journal of Agricultural Economics. 2005;87(5):1200–10.

28. Gordana M-T, Ewa R, Yves SR. Pure and compensated technical efficiency of swedish dairy farms. Agricultural and Food Science. 2016;25(2):111–23.

29. Hanjra MA, Culas RJ. The political economy of maize production and poverty reduction in Zambia: Analysis of the last 50 years. Journal of Asian and African Studies. 2011;46(6):546–66.

30. Herath HMKV, Gunawardena ERN, Wickramasinghe WMADB. The impact of “Kethata Aruna” fertilizer subsidy programme on fertilizer use and paddy production in Sri Lanka. Tropical Agricultural Research. 2013;25(1):14–26.

31. Hosseingholizadeh N, Haghighat J, Mohammadrezaei R. Examining subsidy polices on maize production in Iran (panel data approach). Journal of Agricultural Management and Development. 2014;4(3):171–82.

32. Jaime MM, Coria J, XiangPing L. Interactions between CAP agricultural and agri-environmental subsidies and their effects on the uptake of organic farming. Journal of Agricultural Economics. 2016;98(4):1114–45.

33. JiKun H, XiaoBing W, HuaYong Z, ZhuRong H, Rozelle S. Subsidies and distortions in China’s agriculture: evidence from producer-level data. Journal of Agricultural and Resource Economics. 2011;55(1):53–71.

34. Judzinska A. The influence of direct support under common agricultural policy on farm incomes in Poland. APSTRACT: Applied Studies in Agribusiness and Commerce. 2013;33–7.

35. Kabir MH, Talukder RK. Economics of small scale dairy farming in Bangladesh under the government support programme. Nitis IM, Shin MT, editors. Journal of Animal Sciences. 1999;12(3):429–34.

36. Kallas Z, Serra T, Gil JM. Effects of policy instruments on farm investments and production decisions in the Spanish COP sector. Applied Economics. 2012;44(30):3877–86.

37. Kankwamba H, Kadzamira M, Pauw K. How diversified is cropping in Malawi? Patterns, determinants and policy implications. Food Security. 2018;10(2):323–38.

38. Kannan E. Relationship between agricultural credit policy, credit disbursements and crop productivity: a study in Karnataka. Journal of Agricultural Economics. 2011;66(3):444–56.

39. Kapusta F. Common agricultural policy of the European Union and the changes in Polish agriculture. Acta Scientiarum Polonorum - Oeconomia. 2015;14(1):47–54.

40. Kazukauskas A, Newman C, Sauer J. The impact of decoupled subsidies on productivity in agriculture: a cross-country analysis using microdata. Agricultural Economics. 2014;45(3):327–36.

41. Kirwan BE, Uchida S, White TK. Aggregate and farm-level productivity growth in tobacco: Before and after the quota buyout. American Journal of Agricultural Economics. 2012;94(4):838–53.

42. Lakapunrat N, Thapa GB. Policies, socioeconomic, institutional and biophysical factors influencing the change from rice to sugarcane in Nong Bua Lamphu Province, Thailand. Environmental Management. 2017;59(6):924–38.

43. Lakner S. Technical efficiency of organic milk-farms in Germany - the role of subsidies and of regional factors. Metspalu L, editor. Agronomy Research. 2009;632–9.

44. Lasanta T, Marin-Yaseli ML. Effects of European common agricultural policy and regional policy on the socioeconomic development of the Central Pyrenees, Spain. Mountain Research and Development. 2007;27(2):130–7.

45. Latruffe L, Bravo-Ureta BE, Carpentier A, Desjeux Y, Moreira VH. Subsidies and technical efficiency in agriculture: Evidence from European dairy farms. American Journal of Agricultural Economics. 2017;99(3):783–99.

46. Latruffe L, Desjeux Y. Common agricultural policy support, technical efficiency and productivity change in French agriculture. Review of Agricultural, Food and Environmental Studies. 2016;97(1):15–28.

47. Liang L, Lal R, Ridoutt BG, Zhao G, Du Z, Li L, et al. Multi-indicator assessment of a water-saving agricultural engineering project in North Beijing, China. Agricultural Water Management. 2018;200:34–46.

48. LiPing G, Kinnucan HW, YaoQi Z, GuangHua Q. The effects of a subsidy for grassland protection on livestock numbers, grazing intensity, and herders’ income in Inner Mongolia. Land Use Policy. 2016;302–12.

49. Liu Y, Yao S, Lin Y. Effect of Key Priority Forestry Programs on off-farm employment: Evidence from Chinese rural households. Forest Policy and Economics. 2018;88:24–37.

50. LiYun L, GuanQiao L. Efficiency evaluation of effect of direct grain subsidy policy on performance of rice production. Asian Agricultural Research. 2017;9(4):11–5.

51. Lopez CA, Salazar L, Salvo CP de. Agricultural input subsidies and productivity: the case of Paraguayan farmers. IDB Working Paper Series - Inter-American Development Bank. 2017;

52. Lu WC. Effects of agricultural market policy on crop production in China. Food Policy. 2002;561–73.

53. Malá Z, Červená G, Antoušková M. Analysis of the impacts of Common Agricultural Policy on plant production in the Czech Republic. Acta Universitatis Agriculturae et Silviculturae Mendelianae Brunensis. 2011;59(7):237–44.

54. Manu SA, Fialor S, Issahaku G. Effect of a Food Crop Development Project on livelihood of small-scale maize farmers, Ghana. Journal of Agricultural Sciences. 2012;4(6):395–402.

55. Mary S. Assessing the impacts of Pillar 1 and 2 subsidies on TFP in French crop farms. Journal of Agricultural Economics. 2013;64(1):133–44.

56. Marzec J, Pisulewski A. The effect of CAP subsidies on the technical efficiency of Polish dairy farms. Central European Journal of Economic Modelling and Econometrics. 2017;(3):243–73.

57. Mason NM, Smale M. Impacts of subsidized hybrid seed on indicators of economic well-being among smallholder maize growers in Zambia. (Special Issue: Input subsidy programs (ISPs) in Sub-Saharan Africa (SSA).). Jayne T, Rashid S, editors. Agricultural Economics. 2013;44(6):659–70.

58. Matulova K, Cechura L. Technological heterogeneity, technical efficiency and subsidies in Czech agriculture. Journal of Central European Agriculture. 2016;17(2):447–66.

59. Melece L, Krievina A, Spoge I. Sustainability aspects of bioenergy production: Case of Latvia. In 2016. p. 569–76. Available from: https://www.scopus.com/inward/record.uri?eid=2-s2.0-84994104336&doi=10.5593%2fSGEM2016%2fB53%2fS21.073&partnerID=40&md5=accc5d069392ee96104a9948cdfa7f07

60. Mesike CS. Impact of government agricultural policies on exports of cocoa and rubber in Nigeria. Agricultura Tropica et Subtropica. 2012;45(4):184–8.

61. Morales Olmos V, Siry JP. Economic impact evaluation of Uruguay forest sector development policy. Journal of Forestry. 2009;107(2):63–8.

62. Muncan P, Bozic D. The effects of input subsidies on field crop production in Serbia. Economics of Agriculture. 2013;60(3):585–94.

63. Naglova Z, Gurtler M. Consequences of supports to the economic situation of farms with respect to their size. Agricultural Economics (Zemedelska Ekonomika). 2016;62(7):311–23.

64. Nastis SA, Papanagiotou E, Zamanidis S. Productive efficiency of subsidized organic alfalfa farms. Journal of Agricultural and Resource Economics. 2012;37(2):280–8.

65. Nikola TM, Kehinde O, Mile P. Are agricultural subsidies efficient tool for agricultural sector of the Republic of Macedonia? Journal of Agricultural Science. 2017;23(3):363–9.

66. Nordin M. Does the decoupling reform affect agricultural employment in Sweden? Evidence from an exogenous change. Journal of Agricultural Economics. 2014;65(3):616–36.

67. Nwachukwu IN, Ezeh CI. Impact of selected rural development programmes on poverty alleviation in Ikwuano LGA, Abia State, Nigeria. Journal of Food. 2007;7(5).

68. O’Donoghue EJ, Roberts MJ, Key N. Did the Federal Crop Insurance Reform Act alter farm enterprise diversification? Journal of Agricultural Economics. 2009;60(1):80–104.

69. Okoboi G, Kuteesa A, Barungi M. The impact of the National Agricultural Advisory Services program on household production and welfare in Uganda. Research Series - Economic Policy Research Centre. 2013;

70. Onumah JA, Williams PA, Quaye W, Akuffobea M, Onumah EE. Smallholder cocoa farmers access to on/off-farm support services and its contribution to output in the Eastern region of Ghana. Journal of Agriculture and Rural Development. 2014;4(10):484–95.

71. Padula AD, Santos MS, Ferreira L, Borenstein D. The emergence of the biodiesel industry in Brazil: Current figures and future prospects. Energy Policy. 2012;44:395–405.

72. Pechrova M. Impact of the Rural Development Programme subsidies on the farms’ inefficiency and efficiency. Agricultural Economics (Zemedelska Ekonomika). 2015;61(5):197–204.

73. Peckham JG, Kropp JD. Decoupled direct payments under base acreage and yield updating uncertainty: an investigation of agricultural chemical use. Agricultural and Resource Economics Review. 2012;41(2):158–74.

74. Pilvere I. Payments for less-favoured areas in Latvia. Economics and Rural Development. 2013;9(1):47–55.

75. Possebom V. Free trade zone of manaus: An impact evaluation using the synthetic control method. Revista Brasileira de Economia. 2017;71(2):217–31.

76. Quiroga S, Suarez C, Fernandez-Haddad Z, Philippidis G. Levelling the playing field for European Union agriculture: does the Common Agricultural Policy impact homogeneously on farm productivity and efficiency? Land Use Policy. 2017;179–88.

77. Racul A, Cimpoies D. Some of the financial aspects of agricultural policy in the context of the farm efficiency in the Republic of Moldova. Scientific Papers Series - Management, Economic Engineering in Agriculture and Rural Development. 2012;12(3):159–64.

78. Ragasa C, Chapoto A. Moving in the right direction? The role of price subsidies in fertilizer use and maize productivity in Ghana. Food Security. 2017;9(2):329–53.

79. Ragasa C, Mazunda J, Kadzamira M. The impact of agricultural extension services in the context of a heavily subsidized input system: the case of Malawi. IFPRI - Discussion Papers. 2016;

80. Rizov M, Pokrivcak J, Ciaian P. CAP subsidies and productivity of the EU farms. Journal of Agricultural Economics. 2013;64(3):537–57.

81. Ross M. Leveraging social networks for agricultural development in Africa. Leveraging social networks for agricultural development in Africa. 2017;

82. RunSheng Y, Can L, MinJuan Z, ShunBo Y, Hao L. The implementation and impacts of China’s largest payment for ecosystem services program as revealed by longitudinal household data. Long HL, editor. Land Use Policy. 2014;45–55.

83. Saenz M, Thompson E. Gender and policy roles in farm household diversification in Zambia. World Development (Oxford). 2017;152–69.

84. Seck A. Fertiliser subsidy and agricultural productivity in Senegal. World Economy. 2017;40(9):1989–2006.

85. Serra T, Goodwin BK, Featherstone AM. Agricultural policy reform and off-farm labour decisions. Journal of Agricultural Economics. 2005;56(2):271–85.

86. Sharaunga S, Wale E. The dis-incentive effects of food aid and agricultural policies on local land allocation in developing countries: the case of Malawi. Development Southern Africa. 2013;491–507.

87. ShiWei L, PingYu Z, XiuLi H, ZheYe W, JunTao T. Productivity and efficiency change in China’s grain production during the new farm subsidies years: evidence from the rice production. Custos e @gronegocio. 2015;11(4):106–23.

88. Sianjase A, Seshamani V. Impacts of farmer inputs support program on beneficiaries in Gwembe District of Zambia. Journal of Environmental Issues and Agriculture in Developing Countries. 2013;5(1):40–50.

89. Skreli E, Imami D, Jambor A, Zvyagintsev D, Cera G. The impact of government subsidies on the olive and vineyard sectors of Albanian agriculture. Studies in Agricultural Economics (Budapest). 2015;117(3):119–25.

90. Sokolova E, Kirovski P, Ivanov B. The role of EU direct payments for production decision-making in Bulgarian agriculture. Agriculture and Forestry. 2015;61(4):145–52.

91. Song J, ZhengLin C, Zhao W. The general and structural effect of “four agricultural subsidies” on grain yield: empirical analysis of Chongqing City. Asian Agricultural Research. 2012;4(8):16–8.

92. Takacs Gyorgy K, Takacs I. Changes in cereal land use and production level in the European Union during the period 1999-2009, focusing on New Member States. Studies in Agricultural Economics (Budapest). 2012;24–30.

93. Theriault V, Smale M, Assima A. The Malian fertiliser value chain post-subsidy: an analysis of its structure and performance. Development in Practice. 2018;28(2):242–56.

94. Tleubayev A, Bobojonov I, Gotz L, Hockmann H, Glauben T. Determinants of productivity and efficiency of wheat production in Kazakhstan: a stochastic frontier approach. Discussion Paper, Leibniz Institute of Agricultural Development in Transition Economies (IAMO). 2017;

95. Trnkova G, Mala Z, Vasilenko A. Analysis of the effects of subsidies on the economic behavior of agricultural businesses focusing on animal production. AGRIS On-line Papers in Economics and Informatics. 2012;115–26.

96. Uchida E, Rozelle S, JinTao X. Conservation payments, liquidity constraints, and off-farm labor: impact of the Grain-for-Green program on rural households in China. Journal of Agricultural Economics. 2009;91(1):70–86.

97. Uddin MT, Dhar AR. Government input support on Aus rice production in Bangladesh: impact on farmers’ food security and poverty situation. Agriculture and Food Security. 2018 Feb;7(14).

98. WuSheng Y, Jensen HG. China’s agricultural policy transition: impacts of recent reforms and future scenarios. Journal of Agricultural Economics. 2010;61(2):343–68.

99. XueQin Z, Karagiannis G, Lansink AO. The impact of direct income transfers of CAP on Greek olive farms’ performance: using a non-monotonic inefficiency effects model. Journal of Agricultural Economics. 2011;62(3):630–8.

100. XueQin Z, Lansink AO. Impact of CAP subsidies on technical efficiency of crop farms in Germany, the Netherlands and Sweden. Journal of Agricultural Economics. 2010;61(3):545–64.

101. Yang Z, QingBin W. Impacts of direct government payments on US agriculture: evidence from 1960-2010 data. Wang Y, editor. China Agricultural Economic Review. 2012;4(2):188–99.

102. Ying L. Empirical analysis of China’s direct food subsidy policy based on DEA model: a case study of direct food subsidy policy in Shandong Province. Asian Agricultural Research. 2014;6(9):23–8.

103. Zivenge E, Jesythomas K. Impact of agriculture input support programme on economic benefit in Zimbabwe. Journal of Commerce and Business Management. 2014;7(1):210–4.
